# TCR activation stimulates regulated intramembrane proteolysis of L-selectin by presenilin 1 and localized proteasomal degradation of the cytoplasmic tail

**DOI:** 10.1016/j.jbc.2025.110473

**Published:** 2025-07-10

**Authors:** Owen R. Moon, Andrew C. Newman, Abdullah S. Alanazi, Sophie C. Wehenkel, Katarzyna Gawel-Bęben, Aleksandar Ivetic, David A. Price, Vera Knäuper, Ann Ager

**Affiliations:** 1 Division of Infection and Immunity, School of Medicine, Cardiff University, Cardiff, UK; 2 School of Dentistry, Cardiff University, Cardiff, UK; 3 Faculty of Life Sciences and Medicine, School of Cardiovascular Medicine and Metabolic Sciences, King’s College London, London, UK; 4 Systems Immunity Research Institute, Cardiff University, Cardiff, UK

**Keywords:** ADAM17, CD62L/L-selectin, cell signaling, γ-secretase, imaging flow cytometry, regulated intramembrane proteolysis, T-cell receptor (TCR)

## Abstract

Leucocyte (L)-selectin is essential for mounting protective immunity to pathogens. As well as regulating leucocyte recruitment, it also regulates their activation and differentiation inside tissues thereby shaping local immune responses. The biochemical signals that regulate these diverse functions of L-selectin are poorly understood. Leucocyte activation induces proteolytic shedding of L-selectin ectodomain (ECD) but the impact of ECD shedding on signaling downstream of L-selectin is not known. In T cells, there is substantial overlap between signaling downstream of L-selectin and the T-cell receptor (TCR). Cross-linking of L-selectin stimulates phosphorylation of the cytoplasmic tail and forward signaling *via* the nonreceptor tyrosine kinases Lck and Zap70. Cross-linking of TCR induces phosphorylation-dependent binding of PKC isozymes to L-selectin cytoplasmic tail and PKCα-dependent shedding of ECD. To further understand the role of L-selectin in T cell biology, we used T cells to dissect the cross-talk between L-selectin and physiological TCR activation. We used a combination of imaging flow cytometry and biochemistry to localize L-selectin ECD and intracellular domain (ICD) following engagement of the TCR. We show that following A Metalloproteinase And Disintegrin (ADAM) 17–dependent ECD shedding from the plasma membrane, the ICD-containing transmembrane retained fragment undergoes intramembrane proteolysis by PS1-containing γ-secretase. Subsequent degradation of L-selectin ICD occurs *via* the proteasome in the vicinity of the plasma membrane. Regulated intramembrane proteolysis and rapid degradation of L-selectin ICD following TCR activation suggests that the turnover of L-selectin cytoplasmic tail is an important regulator of T-cell costimulation by L-selectin.

L-, E-, and P-selectins comprise the selectin family of adhesion molecules on leucocytes and endothelial cells that are essential for leucocyte recruitment from the bloodstream to sites of inflammation and immunity (1). Leucocyte (L)-selectin or CD62L is a type 1 transmembrane C-type lectin expressed by the majority of circulating leucocytes. The best understood role for L-selectin on T cells is in recruitment or homing to lymph nodes (LNs) which is required to mount protective immunity to pathogens. L-selectin binds to 6-sulpho sialyl Lewis X glycosylated ligands termed peripheral LN addressin on the luminal and basolateral surfaces of high endothelial venule blood vessels. L-selectin–peripheral LN addressin interactions enable shear stress–dependent tethering and rolling of T cells and subsequent transmigration across the vessel wall to enter the LN parenchyma (2).

Once inside LN, L-selectin has not been considered important for T-cell function. However, *in vitro* studies have reported cross-talk between L-selectin and the TCR. L-selectin engagement stimulates T-cell proliferation (3), superoxide production (4), colony-stimulating factor 1 release (5), lytic activity (6) as well as β1 and β2 integrin-dependent adhesion (7, 8, 9). This may reflect the fact that L-selectin engagement stimulates several signaling pathways that are shared with TCR engagement such as activation of Lck, MAPK, c-abl, ZAP70, Ras, and Rac (4, 5). TCR engagement alone stimulates diacyl glycerol/PKC-dependent phosphorylation of serine residues in the cytoplasmic tail of L-selectin and stable binding of PKC isozymes θ and α which are important regulators of TCR signaling (10). TCR engagement also stimulates ADAM17-dependent shedding of L-selectin ectodomain (ECD) from the T-cell membrane (11). Why L-selectin ECD is shed is not completely understood but it is a widely used marker to distinguish activated from naïve T cells due to loss of cell surface L-selectin. Important clues to the role of L-selectin shedding following TCR activation have come from studies comparing T cells expressing wild type (WT) and shedding-resistant forms of L-selectin. In mouse studies of virus immunity naïve CD8+ T cells unable to shed L-selectin show reduced TCR-dependent clonal expansion inside draining LN after virus inoculation (11), delayed virus clearance by memory CD8+ T cells (12), and reduced clonal expansion in response to TCR engagement *in vitro* (13). Human tumor-specific CD8+ cells expressing a noncleavable form of L-selectin showed reduced lytic activity toward tumor cells which is associated with reduced degranulation and mobilization of CD107a to the T-cell membrane (14). Conversely, in a mouse model of adoptive T-cell cancer therapy, tumor-specific CD8+ T cells unable to shed L-selectin were better able to restrict the growth of solid and disseminated tumors than WT T cells (13). The beneficial effects of T cells unable to shed L-selectin were unrelated to improved recruitment from the bloodstream into tumors or involved LNs but, instead, correlated with early peptide-major histocompatibility complex (MHC) dependent activation of T cells in the tumor microenvironment and in involved lymphoid organs (13). Together, these studies suggest that signals transmitted by L-selectin during TCR engagement regulate biological responses in T cells independently of its homing function. Moreover, L-selectin–dependent signaling is regulated by shedding of the ECD.

What then could be the impact of ADAM17-dependent proteolytic shedding of L-selectin on T-cell activation? Several possibilities can be envisaged which are not mutually exclusive: First, the released ECD competes with ligands on antigen-presenting cells (APCs) and antagonises L-selectin–dependent signals in adjacent T cells. Second, loss of L-selectin ECD immediately shuts off all signaling in T cells that is initiated and sustained by ligand binding or third, the membrane-retained fragment (MRF) of L-selectin undergoes regulated intramembrane proteolysis and the released C-terminal intracellular domain (ICD) propagates TCR-dependent initiated signals.

In this study, we determined the fate of the cytoplasmic tail in TCR-activated T cells using a combination of imaging flow cytometry and biochemical analysis. We report that following TCR clustering and PKC-dependent activation of ADAM17, cleavage of the L-selectin ECD enables intramembrane cleavage of the MRF by presenilin (PS) 1 containing γ-secretase and proteasomal degradation of the ICD in a periplasma membrane location. Our findings suggest that L-selectin cytoplasmic tail is poised for rapid turnover in the membrane of activated T cells and suggests that it actively shapes TCR-driven biological responses.

## Results

### Generation of L-selectin-V5-His to track the cytoplasmic tail in TCR-activated T cells

**Figure 1:**
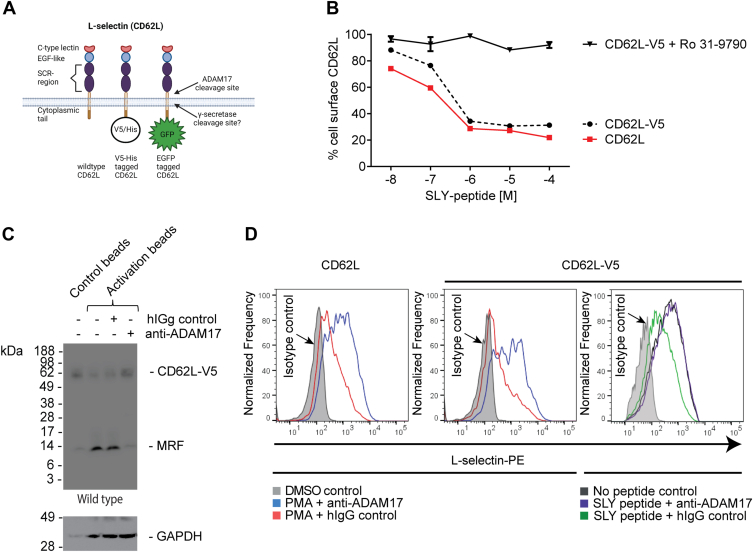
**L-selectin ectodomain shedding is mediated by ADAM17 irrespective of the mode of T-cell activation and intracellular domain tag.***A,* schematic representation of L-selectin expression constructs used in this study. *B,* 868 TCR+ MOLT-3 cells expressing either WT (CD62L) or V5/His-tagged (CD62L-V5) L-selectin were either pretreated with the metalloproteinase inhibitor Ro 31-9790 or dimethyl sulphoxide (DMSO) solvent control prior to engaging the 868 TCR with SLY peptide primed antigen-presenting cells at the indicated concentrations. Data show percentage of cells expressing cell surface L-selectin measured by flow cytometry (mean ± SD, n = 3). Gating strategy shown in Fig. S1. *C,* Western blot analysis of 868 TCR+ MOLT-3 cells pretreated with either an inhibitory ADAM17 antibody or hIgG control antibody and stimulated with T-cell activator beads. Bands for full-length L-selectin-V5/His and the membraneretained L-selectin-V5/His fragment (MRF) are indicated. *D,* flow cytometric analysis of cell surface L-selectin following T-cell activation with either PMA or SLY-pulsed antigen-presenting cells in the presence or absence of inhibitory ADAM17 antibody. ADAM, a metalloproteinase and disintegrin; TCR, T-cell receptor; GFP, green fluorescent protein; MRF, membrane retained fragment; PMA, phorbol 12-myristate 13-acetate.

Our previous work showed that activation of the TCR on CD4+ T cells using MHC class II binding bacterial superantigen *Staphylococcus* enterotoxin B and on CD8+ T cells by peptide MHC1 stimulates ADAM17-dependent ECD proteolysis of L-selectin in human and mouse cells (11). To monitor the accumulation of cleaved fragments of L-selectin after ADAM17 shedding of ECD, we expressed L-selectin tagged with a V5-His tag at the intracellular carboxy terminus (Fig. 1*A*) in MOLT-3 T cells that do not express endogenous L-selectin. This provides a clean background in which to study L-selectin-V5-His using V5-antibodies for Western blotting of cell lysates to detect C-terminal fragments. To study the impact of physiological TCR engagement on L-selectin biology, MOLT-3 T cells expressed 868 TCR which recognises HIV antigenic SLYNTIATK (SLY) peptide bound to HLA-A∗02 on APCs. Transduced MOLT-3 T cell lines coexpressing L-selectin variants and 868 TCR were subjected to rigorous cell biological and biochemical characterization to ensure that C-terminal tagging of L-selectin with the V5-His tag did not interfere with ADAM17-dependent L-selectin ECD shedding. We have reported dose-dependent shedding of non-tail-tagged L-selectin following ligation of 868 TCR by SLY peptide pulsed APC which is completely inhibited by DA1(12) antibody to human ADAM17 (11). Engagement of the 868 TCR with SLY peptide-MHC on APC stimulated dose-dependent shedding of L-selectin-V5-His from the cell surface measured by live-cell imaging (Figs. 1*B*, S1). The dose–response curve was similar to that of non-tagged L-selectin with 10^−6^ M SLY peptide causing maximal loss of L-selectin from the cell surface, which was blocked by the synthetic metalloproteinase (MP) inhibitor Ro 31-9790 (Fig. 1*B*).

To detect ADAM17-generated fragments of L-selectin in cell lysates of TCR-activated cells, we probed western blots with V5 antibody. We used T-cell activator beads coated with anti-CD3/28 instead of peptide-pulsed APCs to avoid diluting T-cell lysates with APC lysates for detection purposes. In the absence of TCR stimulation, a broad band of ∼60 kDa was routinely detected in cell lysates (Fig. 1*C*, control beads). Following TCR activation, the ∼60 kDa band was reduced and a single cleavage product of ∼8 kDa was detected. Inclusion of an inhibitory ADAM17 antibody (D1(A12) (15), retained the ∼60 kDa band and prevented the appearance of the smaller band showing clearly that the latter represents the ADAM17 cleavage product. Together these results show that the ∼60 kDa band represents full length L-selectin-V5-His expressed in MOLT-3 T cells and that it is cleaved by ADAM17 to generate a single V5-tagged, cytoplasmic tail containing species of ∼8 kDa. Although western blotting of whole-cell lysates does not distinguish between membrane and cytoplasmic proteins, we reasoned that by analogy with other type 1 membrane proteins cleaved by membrane inserted ADAMs, the ICD containing product is a membrane retained fragment (MRF). In some experiments, a faint 8 kDa band was detected in nonactivated T cells expressing L-selectin-V5-His (Fig. 1*C*, control beads). This likely reflects the low levels of homeostatic or basal L-selectin shedding known to occur in T cells, which we have shown to be independent of ADAM17 activity (11).

The kinetics and dose response of pharmacological activation of ADAM17 by phorbol 12-myristate 13-acetate (PMA) and shedding of L-selectin from MOLT-3 T cells expressing L-selectin-V5-His were compared directly with cells expressing non-tagged L-selectin (Fig. 1*D*). Using maximal levels of 300 nM PMA (Fig. S1), the kinetics and dose response of L-selectin downregulation from the surface of MOLT-3 T cells were not affected by the V5-His cytoplasmic-tagged tail (Figs. 1*D*, S1). The magnitude of PMA induced downregulation of cell surface L-selectin was similar to that induced by TCR engagement (Fig. 1*D*, middle and right-hand panels). These results show that the V5-His tag does not attenuate ADAM17-dependent shedding of L-selectin following TCR activation by peptide–MHC complexes and anti-CD3/CD28 or PMA.

### TCR stimulation induces both ADAM17 and γ-secretase–dependent proteolysis of L-selectin

**Figure 2:**
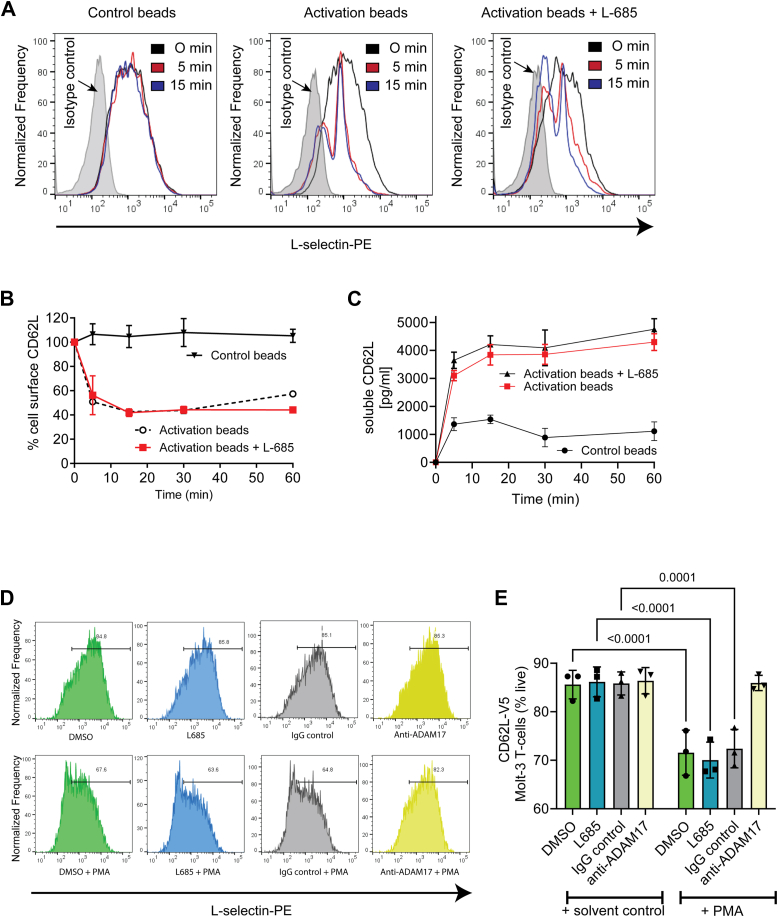
**L-selectin ectodomain proteolysis by ADAM17 is not prevented by** γ**-secretase inhibition.***A–C,* 868 TCR+ MOLT-3 cells expressing V5/His-tagged L-selectin (CD62L-V5) were either pretreated with the γ-secretase inhibitor L-685 or dimethyl sulphoxide (DMSO) solvent control prior to stimulation with either T-cell activator or control beads for the indicated times. Representative histograms showing cell surface expression of L-selectin (*A*), frequency of L-selectin positive cells (*B*), and soluble L-selectin (*C*) are shown. Data are means ± SD, n = 3. *D–E,* 868 TCR+ MOLT-3 cells expressing V5/His-tagged L-selectin (CD62L-V5) were either pretreated with the γ-secretase inhibitor L-685 or blocking ADAM17 antibody and relevant controls prior to stimulation PMA or solvent control. Representative histograms showing cell surface expression of L-selectin (*E*), frequency of L-selectin positive cells. Data are means ± SD, n = 3. Two-way ANOVA with Fisher’s LSD test. TCR, T-cell receptor; ADAM, a metalloproteinase and disintegrin; PMA, phorbol 12-myristate 13-acetate.

Most type I membrane proteins that undergo ECD shedding by ADAMs are susceptible to subsequent proteolytic cleavage within their transmembrane domains by the γ-secretase complex (16, 17). To determine if the MRF of L-selectin generated by ADAM17 shedding of the ECD is susceptible to γ-secretase, we tested ADAM17 activation in the presence and absence of γ-secretase inhibitors L-685 (18) or N-[N-(3,5-difluoro-phenacetyl)-L-alanyl]-S-phenylglycine t-butyl ester (DAPT) (19). We hypothesized that TCR engagement would stimulate rapid (within minutes) sequential proteolysis of L-selectin by ADAM17 and γ-secretase. To understand the role of L-selectin in T-cell function/biology, we wanted to follow the fate of the ICD of L-selectin to determine whether it relocates to another cellular compartment or is degraded. To do this, we used a range of ADAM17-activating stimuli to overcome limitations of conjugate formation between T cells and peptide-pulsed APC, which could dilute out or obscure signals during biochemical analysis. We first tested TCR activation using MOLT-3 T cells transduced with L-selectin-V5-His using T-cell activator beads coated with CD3/CD28 in the presence or absence of γ-secretase inhibitor L-685. Kinetic analysis showed that TCR activation induced detectable loss of L-selectin from the cell surface within the first 5 min and maximal loss at 15 min (Fig. 2, *A* and *B*). Inclusion of L-685 had no effect on TCR-dependent ECD shedding of L-selectin from the T-cell surface (Fig. 2, *A* and *B*) and release of soluble L-selectin (Fig. 2*C*). Analysis of cell-free supernatants confirmed that TCR engagement stimulated rapid release of L-selectin over the first 15 min (Fig. 2*C*). These results show that TCR stimulation activates ADAM17 at early time points, causing rapid cleavage of L-selectin, independent of γ-secretase activity. Although control beads had no effect on cell surface L-selectin, constitutive shedding was evident by release of small quantities of soluble L-selectin (Fig. 2*C*, control beads), which we and others have shown to be independent of ADAM17 (11, 20, 21). ADAM17-dependent shedding of L-selectin induced by PMA was also unaffected by inhibition of γ-secretase activity but blocked by the inhibitory ADAM17 antibody (Fig. 2, *D* and *E*).

**Figure 3:**
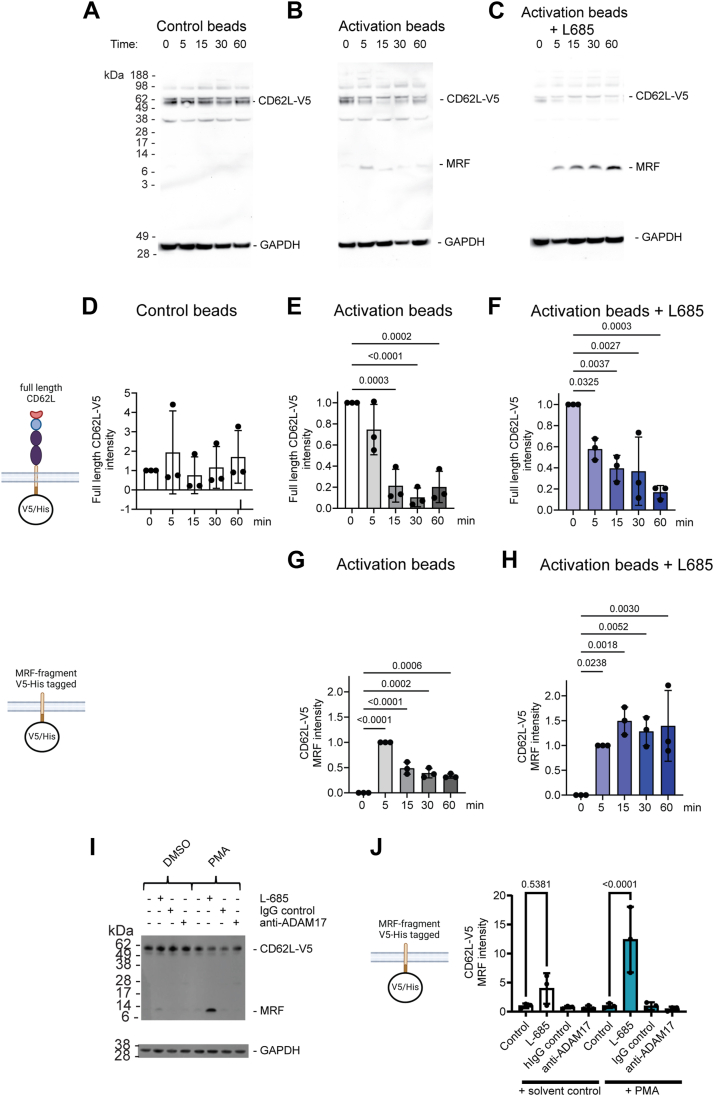
**The membrane-retained fragment of L-selectin is produced by ADAM17 ectodomain shedding and is degraded by** γ**-secretase.** 868 TCR+ MOLT-3 cells expressing V5/His-tagged L-selectin (CD62L-V5) were either pretreated with the protease inhibitors or solvent control prior to stimulation of ADAM17 with T-cell activator or control beads or PMA for the indicated times and analyzed by Western blotting. Representative blots of lysates from control (*A*), T cell activated (*B*), and T cell activated in the presence of γ-secretase inhibitor (*C*). Bands for full-length L-selectin-V5/His and the membrane retained L-selectin-V5/His fragment (MRF) are indicated. Histograms show fold change in full-length L-selectin in control (*D*), T cell activated (*E*), T cell activated in the presence of γ-secretase inhibitor (*F*) and fold change in MRF for T cell activated (*G*), and T cell activated in the presence of γ-secretase inhibitor (*H*). Representative blots of lysates from control and PMA-activated cells in the presence of γ-secretase or ADAM17 inhibitor (*I*). Histograms show fold change in MRF for PMA-activated cells (*J*). Data are means ± SD, n = 3. *D-H,* one-way ANOVA with *post hoc* Tukey test. *J,* two-way ANOVA with Fisher’s LSD test. ADAM, a metalloproteinase and disintegrin; TCR, T-cell receptor; MRF, membrane retained fragment; PMA, phorbol 12-myristate 13-acetate.

Analysis of L-selectin-V5-His by western blotting showed that in control bead treated T cells the 60 kDa doublet representing full-length L-selectin-V5-His was stable over a 60-min time course (Fig. 3, *A* and *D*). In TCR-activated T cells, the 60 kDa band intensity decreased between 5 and 15 min, when it was no longer detectable (Fig. 3, *B* and *E*). The ADAM17 MRF cleavage product was not detectable prior to T-cell activation or in control T cells, however it was detected at 5 min after TCR activation (Fig. 3, *B* and *G*). The MRF decreased over time and was barely detectable at 60 min (Fig. 3, *B* and *G*). In line with observations by flow cytometry, inhibition of γ-secretase did not prevent TCR-induced loss of full-length L-selectin-V5-His over the 60 min time course (Fig. 3, *C* and *F*). However, L-685 stabilized the MRF (Fig. 3, *C* and *H*). These results show that TCR stimulation initiates intramembrane proteolysis of the MRF by γ-secretase. In the absence of L-685, we were not able to detect smaller γ-secretase generated fragment of L-selectin-V5-His in cell lysates. To confirm that the generation of the MRF was independent of the cell stimulation method, we used PMA treatment in the presence or absence of the γ-secretase inhibitor L-685 or ADAM17 inhibitory antibody to determine MRF levels by western blotting, which confirmed findings from TCR-activated cells that L-685 stabilized the MRF and that generation of the MRF is ADAM17-dependent (Fig. 3, *I* and *J*). Endogenous and non-tagged L-selectins behaved similarly in response to PMA in that the MRF was stabilized by γ-secretase inhibition (Fig. S2).

### PS1 but not PS2 caused cleavage of the ADAM17 membrane retained L-selectin product

**Figure 4:**
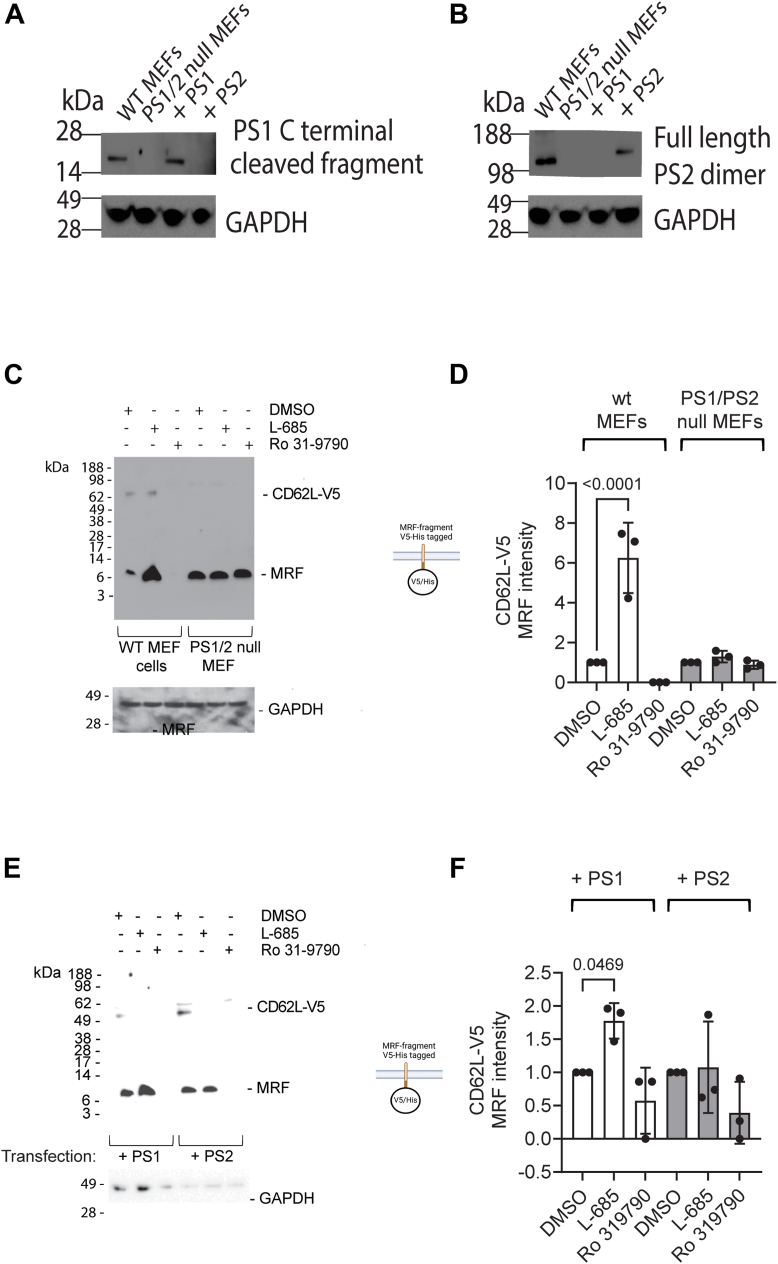
**PS1 mediates intramembrane proteolysis of the ADAM17 product of L-selectin.***A* and *B,* PS1 and PS2 isoform expression in WT, PS1/2 double knockout (PS1/2 null), and PS1/2 null cells expressing PS1 (+PS1) or PS2 (+PS2) validated by immunoblotting. (*C*, *D*) WT or PS1/2 double knockout (PS1/2 null) MEF cells were transiently transfected with L-selectin-V5/His, incubated with L-685 or Ro 31-9790 or DMSO solvent control and analyzed by Western blotting. Representative blots (*C*) and fold changes in MRF in the presence of L-685 and Ro 31-9790 are shown (*D*). *E* and *F*, PS1/2 null MEF cells complemented with PS1 (+PS1) or PS2 (+PS2) were transiently transfected with L-selectin-V5/His and incubated with DMSO, L-685 or Ro 31-9790. Representative blots (*E*) and fold changes in MRF in the presence of L-685 and Ro 31-9790 (*F*) are shown. Results are mean of three independent experiments (n = 3), ± SD. One-way ANOVA with *post hoc* Tukey test. ADAM, a metalloproteinase and disintegrin; DMSO, dimethyl sulphoxide; PS, presenilin; MEF, mouse embryonic fibroblast; MRF, membrane retained fragment.

To confirm intramembrane proteolysis of L-selectin we used a genetic approach of cells deficient in presenilin (PS), the catalytic subunit of γ-secretase. There are two isoforms of presenilin, PS1 and PS2 both of which have been shown to cleave type I transmembrane proteins following cleavage by ADAM17 (17). To study their role in intramembrane proteolysis of L-selectin, we used PS1/2 double null mouse embryonic fibroblast (MEF) cells as well as WT MEFs which express both PS1 and PS2, confirmed by immunoblotting with C-terminal PS1 or PS2 antibodies (Fig. 4, *A* and *B*), (22, 23). WT and PS1/2 null MEFs were transiently transfected with L-selectin-V5-His expression construct and subsequently treated with either γ-secretase inhibitor L-685, MP inhibitor Ro 31-9790, or dimethyl sulphoxide (DMSO) solvent control.

WT and PS null MEFs express relatively high levels of mouse ADAM17, thus activation of ADAM17 activity was not necessary in these cells as cleavage of L-selectin-V5-His was obvious in basal conditions due to limited detection of full-length L-selectin-V5-His (Fig. 4*C*). In WT MEFs L-685 treatment enriched the MRF, while Ro 31-9790 pretreatment prevented its formation. In contrast, L-685 treatment of PS1/PS2 double null cells did not have any effect on the band intensities for the MRF, which was at the same level as the solvent control or Ro 31-9790 treated sample (Fig. 4, *C* and *D*). Next, we used PS1/PS2 double null cells transfected with either PS1 or PS2 (Fig. 4, *A* and *B*) to establish which PS isoform was responsible for MRF cleavage. Following transient transfection with the L-selectin-V5-His expression construct full-length L-selectin was at or below the level of detection in cell lysates of untreated PS1 or PS2 singly expressing MEF cells (Fig. 4*E*). Incubation with the broad spectrum MMP/ADAM inhibitor Ro 31-9790 for 60 min prior to cell lysis had no effect on full-length L-selectin levels. However, the MRF was detected in cell lysates of both PS1 and PS2 expressing MEFs, which was decreased by incubation with Ro 31-9790 confirming its identity as a MP cleavage product.

In PS1-expressing MEFs, there was a significant enrichment of the MRF upon L-685 treatment when compared to DMSO control (Fig. 4, *E* and *F*). In contrast, there was no change in MRF product levels between DMSO and L-685 treated cells in PS2-expressing MEFs (Fig. 4, *E* and *F*). These results indicate that PS1 is responsible for intramembrane proteolysis of the MRF of L-selectin. Full-length L-selectin was not detected following inclusion of the γ-secretase inhibitor L-685 (Fig. 4, *C* and *E*) consistent with its lack of effect on L-selectin-V5-His shedding from the T-cell surface (Fig. 2*B*).

These results suggest that the MRF of L-selectin generated by ADAM17 is subsequently proteolyzed by the intramembrane multisubunit protease complex, γ-secretase containing PS1 but not PS2.

### Fate of L-selectin cytoplasmic tail following ADAM17 and PS1-dependent proteolysis

To determine the subcellular localisation of ADAM17 and γ-secretase generated fragments of L-selectin, we used imaging flow cytometry and analyzed subcellular compartments in MOLT-3 T cells for L-selectin-V5 signals. This technique has the advantage of testing multiple fluorophores on the same cell simultaneously and avoids the requirement to immobilize nonadherent T cells for conventional confocal microscopy. Furthermore, imaging flow cytometry allows high-throughput analysis as every cell is imaged and analyzed. To perform this analysis, we selected PMA as a pharmacologic mimic of TCR/PKC-dependent L-selectin shedding. This approach avoids complications where activator beads or T-cell APC conjugates, would make image analysis more complex. By staining for subcellular compartments (membrane, nucleus, and the lysosome) and the tail of L-selectin, we sought to determine how these fluorescence signals colocalize. The imaging flow cytometry IDEAS software (Cytek) package uses a log-transformed Pearson’s correlation coefficient to evaluate the similarity in the distribution of fluorescence between two probes which is reported as a “similarity score” (24). The similarity score feature is designed for use within user-defined subcellular compartments or “masks”.

**Figure 5:**
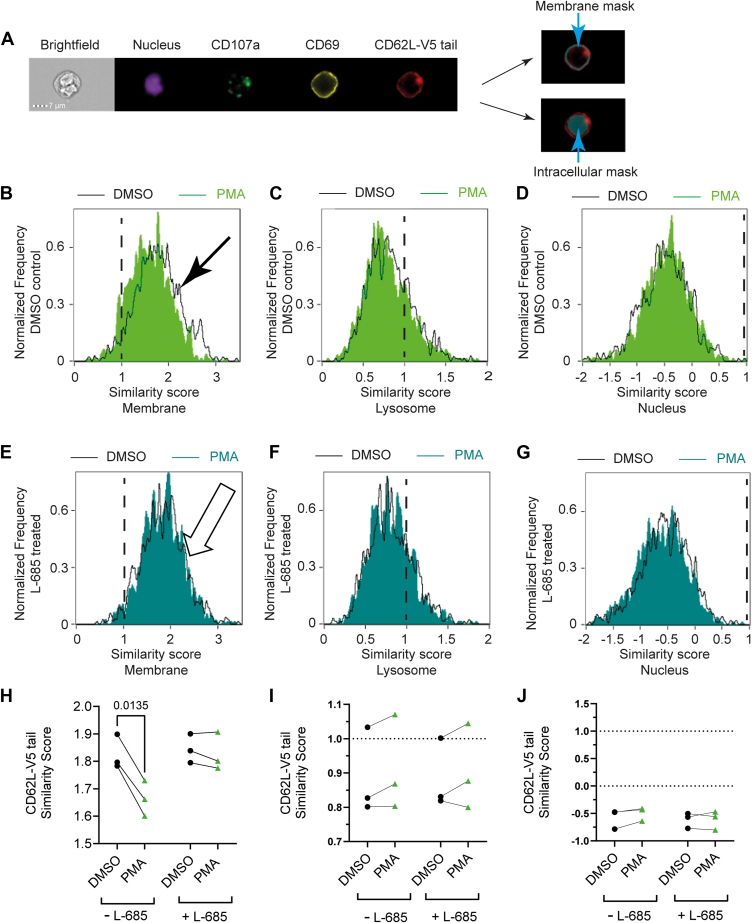
**L-selectin intracellular domain proteolysis by** γ**-secretase causes release from the membrane.** 868 TCR+ MOLT-3 cells expressing V5/His-tagged L-selectin (CD62L-V5) were either pretreated with L-685 or solvent control prior to stimulation of ADAM17 with PMA for 15 min. Live cells were stained for CD69 (membrane), fixed and permeabilized, and then stained for V5 (CD62L tail), CD107a (lysosome) and NucBlue (nucleus), and analyzed by imaging flow cytometry. Triple positive events were AND-gated and assessed for similarity scores between CD62L-V5 and CD69 (membrane), CD62L-V5 and CD107a (lysosome), and CD62L-V5 and NucBlue (nucleus). *A,* representative NucBlue, CD107a, CD69, and CD62L-V5 staining of a single cell and cell masks used to assess similarity scores. *B–D,* representative overlay histograms of similarity scores for L-selectin-V5 tag in the membrane (*B*), lysosome (*C*), and nuclear (*D*) compartments of control (DMSO, *black unshaded*) and activated (PMA, *green shaded*) cells. *Black arrow* in (*B*) indicate shift induced by PMA. *E-G,* representative overlay histograms of similarity scores for L-selectin cyto tail in the membrane (*E*), lysosome (*F*), and nuclear (*G*) compartments of L-685 pretreated control (DMSO, *black unshaded*) and activated (PMA, *cyan shaded*) cells. *White arrow* in (*E*) shows no shift induced by PMA in the presence of L-685. *H–J,* replicate median similarity scores in response to PMA in the absence and presence of γ-secretase inhibitor L-685 in membrane (*H*), lysosomal (*I*), and nuclear (*J*) compartments. Data show mean similarity scores for each replicate without (*black symbols*) and with PMA (*green symbols*), n = 3. Two-way ANOVA with Fishers’s LSD test. ADAM, a metalloproteinase and disintegrin; DMSO, dimethyl sulphoxide; PMA, phorbol 12-myristate 13-acetate; TCR, T-cell receptor.

To determine whether the V5 tag on L-selectin was within the membrane in activated MOLT-3 T cells we searched for an endogenous cell surface marker in MOLT-3 T cells. We stained live MOLT-3 T cells for extracellular CD69 to locate the plasma membrane’s outer leaflet and then fixed and permeabilized the cells and stained for the intracellular V5 tag on L-selectin to define the inner membrane leaflet (Fig. 5*A*, CD69). We plotted the fluorescent signal intensity against the mask number for CD69 and V5 signals and created membrane and intracellular masks (Fig. S3). Within the intracellular mask distinct intracellular compartments, such as the lysosome (CD107a, lysosome marker) or the nucleus (NucBlue signal) were used to assess whether the cytoplasmic tail of L-selectin colocalized to any of these intracellular compartments (Fig. 5*A*). We found CD69 is constitutively expressed in L-selectin-expressing MOLT-3 T cells and CD69 expression levels are unchanged following PMA treatment for up to 15 min (Fig. S3). Therefore, we used CD69 as a membrane marker or these studies. The subcellular location of L-selectin ICD in the absence and presence of γ-secretase inhibitor L-685 was determined 0-, 5-, 10-, and 15-min post-PMA stimulation by determining similarity scores between L-selectin ICD and subcellular compartments (membrane, nucleus, and the lysosome).

Cells positive for CD69, CD107a, and the L-selectin tail (by V5-His tag staining) relative to the isotype control (Fig. S4) were “AND-gated” and triple positive events were assessed for their median similarity scores between pairs of markers within the relevant cellular compartments. Representative staining (Fig. 5*A*) and normalized frequencies plotted against similarity scores for the 15-min time point with PMA (activated) or DMSO control (unactivated) T cells are shown (Fig. 5, *B*–*D*, control: black unshaded histograms; PMA: green shaded histograms). Similarity scores for L-selectin-ICD V5-signal and the CD69 membrane signal in the absence of activation were well above 1 (Fig. 5*B*, black histogram) and averaged 1.84 (Fig. 5*H*, black symbols). This supports the findings of conventional flow cytometry where full-length L-selectin, that is, before the ECD is shed, is detectable at the surface of MOLT-3 T cells under basal conditions (Figs. 1 and 2). In comparison, similarity scores for L-selectin-ICD V5-signal and the lysosome ranged from 0.8 to 1.0 in unactivated cells (Fig. 5*C*, black histogram, Fig. 5*I,* black symbols). Furthermore, similarity scores for L-selectin-ICD V5-signal and the nucleus in unactivated cells were consistently less than 0 (Fig. 5*D*, black histogram; Fig. 5*J* black symbols). Together these findings show that L-selectin prior to cell activation is preferentially located in the plasma membrane of MOLT-3 T cells.

The similarity score between the L-selectin tail and CD69 in the membrane mask decreased at 15 min after PMA addition (Fig. 5*B*, green shaded histogram, black arrow). The similarity score was reduced to 1.66, although it remained above 1 (Fig. 5*H*, green symbols). PMA had no effect on similarity scores between L-selectin-ICD V5-signal and the lysosomal compartment (Fig. 5, C and I;<1) nor in similarity scores between L-selectin-ICD V5-signal and the nuclear compartment (Fig. 5, D and J;<0), suggesting that the cleaved L-selectin-ICD V5 MRF does not colocalize with either the lysosomal or nuclear compartments. Inclusion of the γ-secretase inhibitor L-685 stabilized similarity scores between L-selectin-ICD V5-signal and the plasma membrane in the presence of PMA at 1.84 which was similar to solvent control levels (Fig. 5*E*, cyan shaded histogram, white arrow; Fig. 5*H*, green symbols) suggesting that γ-secretase activity is essential for loss of ICD from the plasma membrane. L-685 had no effect on colocalization of L-selectin-ICD V5-signal with either lysosomal or nuclear markers in the presence of PMA (Fig. 5, I and J).

Interestingly, staining for V5 tag revealed distinct intracellular “deposits” or spots of L-selectin-V5 signal (Fig. 5*A*). Some evidence for colocalization between L-selectin-ICD V5-signal and CD107a was seen in a small subpopulation of cells which had similarity scores >1 (Fig. 5*C*). Although we found no increase in similarity scores between L-selectin-ICD V5-signal and CD107a following PMA treatment, it is possible that L-selectin-ICD moves to an intracellular compartment which is not detected by CD107a staining. We therefore determined if the loss of ICD colocalization with the membrane in response to PMA was accompanied by an increase in the number of spots per cell as the L-selectin-V5 cytosolic tail is cleaved and potentially moves away from the membrane and into a distinct intracellular compartment. However, no increase in the number of intracellular spots was seen at 15 min post-PMA addition in the presence or absence of L-685 (Fig. S4). Intracellular spots may represent the high levels of Golgi-associated full-length L-selectin produced in lentivirally transduced T cells.

### Degradation of L-selectin ICD following ADAM17 and PS1 proteolysis

We hypothesized that the loss of L-selectin tail staining from the membrane and lack of accumulation in an intracellular compartment was due to degradation of the tail, although other possibilities exist. To address this hypothesis, a lentiviral vector encoding human L-selectin tagged with GFP instead of V5 (Fig. 1*A* (25) was used to transfect the parental L-selectin negative MOLT-3 T cell line and these cells were treated with PMA for 15 min prior to imaging flow cytometry.

**Figure 6:**
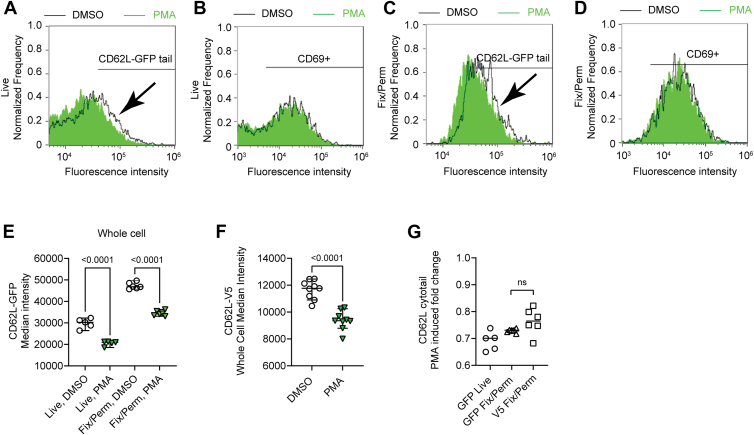
**L-selectin intracellular domain proteolysis by** γ**-secretase causes release from the membrane and degradation.** MOLT-3 cells expressing CD62L-GFP (A-E) were incubated with PMA or DMSO solvent control for 15 min and stained for membrane CD69. CD69 and GFP were analyzed by imaging flow cytometry in live cells and following fixation and permeabilization. *A–D,* representative overlay histograms showing whole-cell L-selectin-GFP or CD69 fluorescence signals in the presence (*green shaded*) and absence (*black unshaded*) of PMA in live (*A* and *B*) and fixed/permeabilised cells (*C* and *D*). *Black arrows* in (*A*) and (*C*) indicate shifts induced by PMA. *E,* median fluorescence whole-cell intensities of GFP in response to PMA in replicate experiments. Results show data points and bars show means ± SD, n = 6. One-way ANOVA with Tukey’s multiple comparisons test. *F,* 868 TCR+ MOLT-3 cells expressing CD62L-V5 were incubated with PMA or DMSO solvent control for 15 min and live, focused, single cells analyzed for whole-cell median fluorescence intensity of L-selectin cyto tail using V5 staining. Data points show median fluorescence whole-cell intensities of V5 in response to PMA. Bars represent means ± SD, n = 6. Unpaired *t* test. *G,* fold change in CD62L cyto tail (GFP or V5) whole-cell intensities in response to PMA. Results show data points and bars show means ± SD, n = 6. One-way ANOVA with Tukey’s multiple comparisons test. ns, not significant. DMSO, dimethyl sulphoxide; PMA, phorbol 12-myristate 13-acetate; TCR, T-cell receptor.

PMA treatment was sufficient to reduce whole-cell L-selectin-GFP fluorescence in live cells (Fig. 6*A*) while CD69 levels remained stable (Fig. 6*B*). For PMA-treated cells, the GFP median intensity decreased significantly regardless of whether the cells were live or fixed and then permeabilized (Fig. 6, *A* and *C*, compare filled green histograms to black lined histograms). The live cells appeared dimmer by median fluorescence intensity (MFI) measurements relative to cells fixed and then permeabilized, but this was due to the presence of GFP^dim^ cells, and not to the loss of GFP^bright^ cells (Fig. 6, *A* and *C*). PMA induced a ∼30% loss in L-selectin-GFP signal in live cells showing clearly that L-selectin ICD signal is reduced following PMA activation as found for V5-tagged ICD (Fig. 6*E*). The loss of L-selectin-GFP signal in fixed/permeabilized cells was similar (Fig. 6*E*). As shown in the representative histograms, CD69 remained stable following PMA treatment in both live and fixed/permeabilized cells and therefore could be used as a membrane marker in experiments with this MOLT-3 cell line expressing GFP-tagged L-selectin (Fig. 6, *B* and *D*). V5-His–tagged L-selectin behaved similarly in that whole-cell fluorescence intensity decreased in response to PMA treatment (Fig. 6*F*) and the fold changes in GFP and V5 fluorescence in fixed and permeabilized cells were not different (Fig. 6*G*).

Together, these findings support the conclusion that loss of L-selectin ICD tail from the membrane is not due to leakage from the cells during fixation and permeabilization. The fact that both V5 and GFP signals decrease suggest that loss of V5 signal is not simply due to a binding partner preventing access of antibody to the tail. Rather, the PMA-induced loss in fluorescence is likely due to degradation of the MRF.

### Proteasomal degradation of L-selectin ICD following TCR activation

Our findings thus far support the hypothesis that TCR induced activation of ADAM17 caused cleavage of the ECD of L-selectin, the MRF undergoes proteolysis by γ-secretase and the ICD is subsequently degraded. Imaging flow cytometry showed that the L-selectin ICD is rapidly lost from the plasma membrane as early as 15 min after TCR stimulation and provided evidence of L-selectin ICD degradation. To confirm experimentally that the L-selectin ICD is degraded we used western blotting and determined the impact of the proteasome, endolysosome, and autophagosome on L-selectin ICD in TCR-stimulated cells. We used MOLT-3 T cells expressing the 868 TCR and V5-His tagged L-selectin and stimulated the cells with SLY cognate peptide pulsed APCs to activate the TCR leading to sequential proteolysis of L-selectin by ADAM17 and γ-secretase. 868 TCR+, L-selectin-V5+, MOLT-3 T cells incubated with the APCs pulsed with NLV peptide were used as negative controls. Although C1R APCs express endogenous L-selectin, it is not tagged with V5 and therefore not detected by western blot analysis with the V5 antibody.

**Figure 7:**
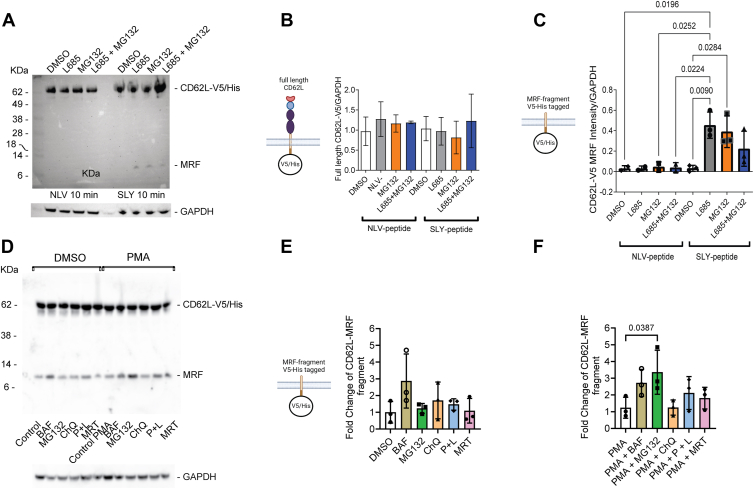
**The membrane-retained fragment of CD62L generated by TCR activation is degraded by γ-secretase and proteasome.** 868 TCR+ MOLT-3 cells expressing CD62L-V5 were pretreated with the indicated protease inhibitors or solvent control prior to activation by SLY or NLV peptide-pulsed antigen-presenting cells (*A*–*C*) or PMA (*D*–*F*). Cells were lysed and immunoblotted for L-selectin's V5/His-tagged cytosolic tail and GAPDH as a control for protein loading. *A,* a representative western blot for full-length CD62L-V5/His and MRF and GAPDH as loading control. *B,* quantification of full-length L-selectin relative to GAPDH control and presentation as fold change following peptide treatment. *C,* quantification of MRF relative to GAPDH control and presentation as fold change following peptide treatment. *D,* a representative western blot for full-length and L-selectin tail containing MRF following pretreatment with the indicated lysosomal and autophagy inhibitors prior to incubation with PMA. Results show fold change in L-selectin MRF in the absence (*E*) and presence of PMA. *E* and *F,* results are mean of three independent experiments (n = 3), ± SD. One-way ANOVA with Tukey’s multiple comparisons test. Lysosomal inhibitors: BAF, bafilomycin; ChQ, chloroquine; P + L, pepstatin and leupeptin. Autophagy inhibitor: MRF, MRT68921. MRF, membrane-retained fragment; PMA, phorbol 12-myristate 13-acetate; TCR, T-cell receptor.

Samples were analyzed 10 min after activation since the MRF is rapidly lost from whole-cell lysates by 15 min (Fig. 2). At this early time point, changes in full-length L-selectin were not detectable (Fig. 7, *A* and *B*). As found using polyclonal T-cell activation using CD3/28 activator beads (Fig. 2), ADAM17 activation by peptide-MHC engagement of TCR did not generate a detectable MRF unless γ-secretase activity was inhibited (Fig. 7, *A* and *C*), whereas the control peptide NLV did not stimulate ADAM17-dependent shedding of L-selectin and therefore MRF was not detected even in the presence of L-685 (Fig. 7, *A* and *C*). We tested the proteasome inhibitor MG132 to determine if this would stabilize a smaller L-selectin fragment representing the ICD released by γ-secretase. T cells were pretreated with MG132 and then stimulated with peptide MHC carrying either activating SLY or NLV control peptide in the presence or absence of MG132. MG132 enriched an L-selectin ICD fragment of around 8 kD in SLY but not in NLV control peptide-treated T cells (Fig. 7, *A* and *C*). Interestingly the L-selectin ICD fragment stabilised by proteasomal inhibition was the same size as the MRF stabilised by L-685 and a smaller fragment predicted to represent the γ-secretase generated ICD was not detected. The effects of either MG132 or L-685 alone to prevent loss of the MRF were equivalent (Fig. 7*C*). To determine if L-685 and MG132 had additive effects on the MRF, T cells were pretreated with both inhibitors and TCR stimulated with peptide-MHC as above. The combination of L-685 and MG132 enriched an L-selectin ICD fragment of around 8 kD in SLY but not in NLV-treated T cells (Fig. 7*A*). However, this was not significantly different from T cells treated with vehicle control DMSO alone (Fig. 7, *A* and *C*). There was no evidence for endolysosomal degradation or autophagic recycling of L-selectin MRF since inclusion of lysosomal and autophagy inhibitors did not stabilize the L-selectin MRF in either unstimulated or PMA-stimulated cells (Fig. 7, *D*–*F*). Together these findings demonstrate that the γ-secretase generated ICD of L-selectin undergoes rapid proteasomal degradation in close proximity to the plasma membrane.

## Discussion

**Figure 8:**
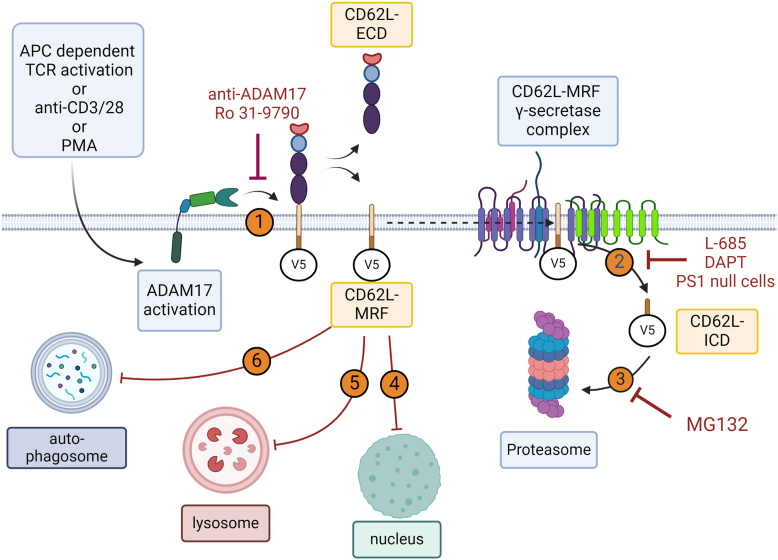
**TCR activation stimulates regulated intramembrane proteolysis of cell surface L-selectin by presenilin 1 and localized proteasomal degradation of the cytoplasmic tail.** Based on a combination of imaging and biochemical experiments using genetic manipulation and pharmacological inhibitors, we propose that cell surface L-selectin undergoes the following sequence of processing steps in T cells following engagement of the TCR. *1,* PKC-dependent proteolysis of L-selectin ectodomain (CD62L-ECD) by ADAM17 and release of C62L-ECD from the T-cell surface. *2,* the membrane-retained fragment (CD62L-MRF) generated by ADAM17 forms a complex with γ-secretase and undergoes intramembrane cleavage by the PS1 subunit of γ-secretase. *3,* the cytoplasmic tail (CD62L-ICD) generated by PS1 undergoes proteasomal degradation. The CD62L-MRF was not translocated to (*4*) the nucleus for downstream signaling, (*5*) the lysosome for degradation or (*6*) the autophagosome for recycling. Proteasomal degradation of L-selectin cytoplasmic tail occurred within 15 min of T-cell activation. ADAM, a metalloproteinase and disintegrin; TCR, T-cell receptor; ICD, intracellular domain.

In this study, we have used a combination of subcellular imaging, genetic, and pharmacological inhibition as well as biochemical analysis to follow the fate of the cytoplasmic tail of cell surface L-selectin in T cells following engagement of the TCR. Our studies show that cell surface L-selectin is proteolyzed exclusively by ADAM17, the L-selectin fragment remaining in the membrane is subsequently cleaved by PS1 and the ICD undergoes proteasomal degradation in or adjacent to the plasma membrane (Fig. 8). We have already demonstrated that ADAM17-dependent shedding of L-selectin regulates clonal expansion of T cells, since T cells unable to shed L-selectin following engagement of the TCR show markedly reduced T-cell proliferation *in vitro* and *in vivo*. The demonstration here that proteasomal degradation of L-selectin ICD is a consequence of shedding raises the possibility that proteasomal turnover of L-selectin ICD actively regulates T-cell function.

The cytoplasmic tail of L-selectin plays an active role in controlling shedding of the ECD. Constitutive binding of calmodulin to hydrophobic residues Ile^352^ and Ile^354^ in L-selectin ICD causes resistance to L-selectin shedding (26, 27). Phosphorylation at Ser^364^ causes dissociation of calmodulin and facilitates ECD proteolysis (25). Additionally, differential binding of ezrin and moesin to Arg^357^ and Lys^362^ in the ICD regulate shedding of L-selectin (28, 29). In our studies, L-selectin was fused to a carboxy-terminal V5-His tag, which potentially could have hindered interaction with these protein binding partners abrogating proteolysis. Therefore, we confirmed that the V5-His–tagged L-selectin was susceptible to proteolysis after TCR activation. Our data showed loss of cell surface expression for both V5-His and non-tagged L-selectin after TCR activation, which was blocked by Ro 31-9790 and the inhibitory anti-ADAM17 antibody D1(A12). TCR-activated cells also showed loss of full-length L-selectin and generation of the MRF by immunoblot analysis which was blocked by the anti-ADAM17 antibody (D1(A12)). Pharmacological activation of ADAM17 by PMA showed similar dose responses and time courses for ECD shedding of non-tagged and tagged L-selectin. The V5-His tag did not interfere with γ-secretase–dependent degradation since endogenous, non-tagged and tagged L-selectin MRF were both enriched by treatment with γ-secretase inhibitors. Collectively, our data confirmed that the C-terminal V5-His tag of L-selectin did not interfere with ADAM17 or γ-secretase–dependent proteolysis after TCR activation. This agrees with previous studies where L-selectin GFP undergoes PMA-induced ECD shedding (25).

Flow cytometry and immunoblot analysis showed that activation of the TCR caused ECD proteolysis of L-selectin independently of γ-secretase activity since the MRF and released ECD were detectable within the first 10 to 15 min in the absence or presence of γ-secretase inhibitors. These results confirmed that after TCR stimulation, ADAM17 sheds L-selectin at the ECD before further proteolytic processing. Immunoblot analysis showed that the ADAM17 product of L-selectin detected at 5 min post-TCR stimulation decreased from 5 min to 60 min, which was blocked by the γ-secretase inhibitor L-685. Together, these results showed that TCR stimulation induces sequential proteolysis of full-length L-selectin first by ADAM17 and then by γ-secretase.

PS is the catalytic subunit of the γ-secretase multiunit complex (30). γ-secretase assembly into a functional complex occurs in a stepwise manner in the endoplasmic reticulum, where PS undergoes endoproteolysis between residues Thr^291^ and Ala^299^ to generate a 30 kDa N-terminal fragment and a 17 kDa C-terminal fragment which form the catalytically active enzyme (31, 32). There are two isoforms of PS, PS1 and PS2, which show some substrate selectivity. For instance, APP and Notch are proteolyzed by both PS1 and PS2 (22, 33, 34), whereas ErbB4 is selectively proteolyzed by PS1 (35). To determine whether the MRF product of L-selectin was cleaved by PS1 and/or PS2, PS-deficient MEF cells were complemented with PS1 and/or PS2. We found that PS1, but not PS2, expression was required for proteolysis of the L-selectin MRF product. Further studies will be required to determine if PS1 is constitutively active in T cells or requires TCR engagement for endoproteolytic activation.

To dissect the role of L-selectin shedding in TCR induced signaling we followed the fate of L-selectin ICD generated by γ-secretase. L-selectin ICD was not detectable as a distinct molecular species separable from the larger ICD containing MRF by western blotting. ICDs of other type I transmembrane proteins released by γ-secretase are often not readily detectable by western blot analysis. Pretreatment of cells with proteasomal inhibitors for 5 to 24 h prior to cell lysis enabled detection of ICDs released from full-length proteins, such as Notch in epithelial cells (36), LDLR in HEK and neuroglioblastoma cells (37, 38), MHC class I in CHO cells (39), and protocadherin in HEK cells (40). Contrastingly, degradation of the ICD of APP is not blocked by proteasome or lysosomal inhibitors (41). Instead, the APP ICD is rapidly cleared by the insulin degrading enzyme, a cytoplasmic MP that proteolyzes small polypeptides (42). Studies have overcome degradation of endogenous ICDs by overexpressing cDNAs encoding the ICDs of APP (AICD) and Notch (NICD) or constitutively active Notch and demonstrated translocation of ICDs to the nucleus where these fragments act as transcriptional factors. Overexpressed APP ICD in the cytoplasm forms a multimeric complex with Fe65 nuclear adaptor protein and the histone acetyltransferase Tip-60 inducing transcription of APP and other genes (43, 44). Comparably, ICD of constitutively active Notch binds to the transcription factor CSL (CBF1/RBPJκ, Su(H), LAG-1) in the nucleus and binds to DNA (45). In other studies, phosphorylated Notch ICD is ubiquitinated and degraded by the proteasome in the nucleus (46, 47). Based on these findings, we hypothesized that the L-selectin ICD is either rapidly degraded and/or trafficked to a subcellular compartment which was not solubilized during cell lysis and therefore could not be detected by western blotting.

Imaging flow cytometry was used to track the subcellular location of cytoplasmic tail of L-selectin in T cells following activation of ADAM17. To achieve this, a method for distinguishing plasma membrane from intracellular cytoplasm in T cells was required. Antibodies that bind to the ECD of L-selectin and CD69 in the outer lipid bilayer and antibodies to V5, which binds to the tail of CD62L-V5 in the inner lipid bilayer were used to distinguish plasma membrane from cytoplasm in 868 TCR+ MOLT-3 cells. Interestingly, CD69 which was constitutively expressed by 868 TCR+ MOLT-3 cells was not affected by PMA, whereas L-selectin ECD was shed and therefore CD69 was used as a membrane marker. A range of parameters were measured including whole-cell MFI values, as in conventional flow cytometry, as well as the MFI in the membrane. In addition, the lysosomal compartment was defined using CD107a and the nucleus with NucBlue, which allowed us to quantify colocalization of L-selectin with the membrane and distinct intracellular compartments as a similarity score between specific fluorophore pairs.

Imaging flow cytometry showed clearly that the ECD of L-selectin was shed from the plasma membrane/cell surface within 15 min following activation of ADAM17, as demonstrated by conventional flow cytometry. The similarity of fluorescence associated with the L-selectin tail and the membrane marker CD69 significantly decreased at 15 min, indicating that the V5-tagged tail is lost from the plasma membrane, which was prevented by inclusion of the γ-secretase inhibitor L-685. This indicates that the γ-secretase complex is colocated where its substrate, the ADAM17-dependent MRF is generated. V5 detection in lysosomal and nuclear compartments did not change following γ-secretase activation suggesting that the V5-tagged tail did not relocate to these compartments. To determine if loss of V5-tail signal was due to degradation, we used L-selectin-GFP, which enabled analysis of live cells and avoided fixation and permeabilization processes required to detect the V5 tag. In live cells, GFP signal was reduced by ADAM17 activation showing that loss of L-selectin tail is due to degradation. In fixed and permeabilized cells ADAM17 activation also induced a loss of L-selectin-GFP confirming that L-selectin-GFP behaves similarly to L-selectin-V5. The reduction in GFP- and V5-tail signals was similar. Together these results demonstrate that loss of V5 signal from the membrane is not an artefact due to procedures used for detection but due to degradation.

To determine whether the L-selectin tail is degraded by the proteasome, we analyzed the effect of MG132 on L-selectin-V5 fragments in TCR-activated MOLT-3 cells by western blotting. Inclusion of MG132 enriched a single band of 8 kDa in SLY peptide-activated T cells, which was a similar in size to the MRF seen in γ-secretase inhibited cells. This raised the possibility that the MRF undergoes direct proteasomal degradation that may not require prior release of ICD by γ-secretase. To determine if MRF undergoes proteasomal degradation when γ-secretase is blocked, we used a combination of MG132 and L-685. Interestingly, the combination of MG132 and L-685 did not further enrich 8 kDa fragment levels over that with either MG132 or L-685 alone. Instead, MG132 and L-685 in combination had a reduced effect and although enrichment of the 8 kDa fragment was seen it was not significantly different from control cells. This could be because the inhibitors interfere with each other’s activity which will require further investigation. There was no evidence for removal of the ICD-containing MRF *via* lysosomal or autophagic pathways. There was also no evidence of relocation of L-selectin ICD to the nucleus. However, Notch ICD is below the level of detection in the nucleus of intact cells and requires overexpression to detect its transcriptional activity (45). The L-selectin ICD contains a conserved C-terminal PY motif. Studies have shown that C-terminal PY motifs act as nuclear import signals for Hrp-1 and Nab-2 (48) so could potentially also regulate nuclear import of L-selectin ICD. Further studies will therefore be required to determine if L-selectin ICD has transcriptional activity.

It is curious that no new proteolysis product corresponding to the γ-secretase cleaved ICD could be observed either by western blotting or by imaging flow cytometry. This raises the possibility that the cleaved ICD stays associated with MRF in the membrane and is degraded in a membrane-associated proteasome. It is also possible that a cleaved ICD in TCR-activated cells is phosphorylated on Ser^364^ and Ser^367^ (25) and the increase in negative charge results in comigration of phosphorylated ICD with the MRF on SDS-PAGE analysis.

Proteasomal degradation of L-selectin ICD would require prior ubiquitination by an E3 ubiquitin ligase. Interestingly, basal turnover of full-length L-selectin in T cells is not regulated by proteasomal degradation (49). However, K5, an E3 ubiquitin ligase in Kaposi sarcoma herpes virus has been shown to downregulate full-length L-selectin in lymphocytes. L-selectin downregulation is dependent on ubiquitination of lysines in the ICD of L-selectin but is not dependent of ECD shedding. Moreover, KSHV E3 ubiquitin ligases target full-length membrane proteins for internalisation and endolysosomal degradation (50, 51). Degradation of L-selectin MRF was not stabilised by lysosomal inhibition and therefore endolysosomal degradation of internalized MRF is unlikely. Further studies will be required to determine whether the L-selectin ICD generated following TCR activation is a cognate substrate for the ubiquitin-proteasomal system and, if so, which of the >600 E3 ubiquitin ligases in the human genome is involved (52).

The biochemical relevance of PS1 proteolysis of the ADAM17 generated L-selectin MRF and subsequent proteasomal degradation of the cytoplasmic tail in activated T cells are currently unknown. ADAM17-dependent shedding of L-selectin plays a significant role in controlling T cell–dependent immunity to tumours (13) as well as to viruses (12, 53). It is known that L-selectin shedding controls vascular recruitment and homing of activated T cells to sites of immune activity (54). In addition, L-selectin shedding also controls T-cell proliferation (11) and lytic activity (14) which are both processes that are not related to homing.

Interestingly, imaging studies have shown that TCR is located on microvilli where L-selectin is selectively localized (55). This raises the possibility that TCR and L-selectin colocation facilitates cross-talk between these two receptors. It has been shown that PKCα binds to the cytoplasmic tail of L-selectin (10) and that PKCα activity controls L-selectin shedding in activated T cells (56). It is possible that turnover of the cytoplasmic tail of L-selectin is a mechanism for removing PKCα or other nonreceptor kinases and adaptor proteins from the TCR to coordinate TCR signaling for efficient immune cell immunity. Further studies of the proteome in TCR-activated T cells where L-selectin processing by ADAM17, PS1, and the proteasome are regulated will be required to fully understand the full impact of L-selectin shedding on T function at sites of immune activity.

## Experimental procedures

### L-selectin expressing T cells

Human L-selectin negative MOLT-3 acute lymphoblastoid T cells (American Type Culture Collection CRL-1552) were sequentially transduced to coexpress an HIV-1 Gag SLYNTVATL-specific TCR (868) with either human L-selectin-V5/His, non-tagged L-selectin, or L-selectin-GFP (25) using methods described previously (11). T cells coexpressing 868 TCR and L-selectin were sorted and grown in complete RPMI 1640 medium containing 100 IU penicillin/ml, 100 μg/ml streptomycin, 1 mM sodium pyruvate, and supplemented with 10% foetal calf serum (FCS) (R10). Jurkat cells (American Type Culture Collection, Clone E6.1) expressing 868 TCR generously provided by John Bridgeman were grown in R10. MEF deficient in ADAM17 (57) were a kind gift of Carl Blobel (HSS Research Institute), and MEF cells deficient in PS1 and PS2 (PS1/PS2 null) stably expressing exogenous PS1 (+PS1) or PS2 (+PS2) (23) were a kind gift of B. De Strooper (KUL). MEF cells were grown in complete Dulbecco's modified Eagle's media containing 100 IU penicillin/ml, 100 μg/ml streptomycin, 1 mM sodium pyruvate and supplemented with 10% FCS (D10). For transient transfection, 2.5 × 10^5^ MEF cells/well were seeded in 1 ml D10 in a 24-well plate and grown to 70% confluence at 37 °C for 24 h. Transfection mix containing 8 μl FuGENE 6, 2 μg pcDNA5 expression vector for V5-His–tagged WT human L-selectin and 90 μl Opti-MEM medium/well was incubated for 35 min at room temperature (RT), added to MEFs, and incubated at 37 °C for 24 h before further experimentation.

### L-selectin shedding stimuli

#### Polyclonal T-cell activation using anti-CD3/28

A total of 80 μl Dynabeads Human TCR-activator CD3/CD28 beads (Cat. No 11131D, Thermo Fisher Scientific) or equivalent numbers of Dynabeads coated with mouse anti-sheep IgG as control (Cat. No 11031, Thermo Fisher Scientific) were washed in PBS, magnetically pelleted and incubated with 1.3 × 10^6^ MOLT-3 T cells in 100 μl complete RPMI 1640 supplemented with 1% FCS (R1) for 5 to 60 min in triplicate 96-well U-bottomed plates. After TCR activation, 2.5 × 10^5^ MOLT-3 T cells were prepared for flow cytometry and the remaining MOLT-3 T cells were collected by centrifugation, lysed in RIPA or Laemmli buffer for western blot analysis. Cell-free supernatants were collected for ELISA.

#### TCR activation by peptide-MHC

TCR-induced L-selectin shedding in MOLT-3 cells was examined in response to HLA-A2^+^ C1R B cells pulsed with cognate SLYNTVATL (SLY) peptide or nonactivating NLVPMVATV (NLV) peptide as control (Peptide Synthetics). MOLT-3 cells and C1R cells were rested overnight at 0.9 × 10^6^ cells/ml in RPMI 1640 plus 2% FCS (R2). The following day, C1R cells were resuspended at 0.5 × 10^6^ cells/ml in the presence of SLY (10^−7^–10^−4^ M) or NLV (10^−5^ M) peptides in R2 and plated at 0.5 × 10^5^ cells/well in triplicate in 96-well U-bottomed plates. Cells were incubated for 1 h at 37 °C and washed in R2. MOLT-3 cells were resuspended at 1.5 × 10^6^ cells/ml and added to C1R cells at a ratio of 3:1. The plates were incubated for 5 to 60 min at 37 °C. Cells were washed in ice-cold PBS, pelleted and stained for flow cytometry, or lysed for western blot analysis. Cell-free supernatants were stored for ELISA analysis at −20 °C.

#### Cell activation using PMA

Triplicate wells of MOLT-3 or Jurkat T cells (2.5 × 10^5^ cells/well in100 μl R2 in 96-well U-bottomed plates) or MEFs (2.5 × 10^5^ cells/100 μl D2 in 24-well plates) were stimulated with 300 nM PMA by addition of 3 μl of 10 μM stock PMA in DMSO) or equivalent amount of DMSO per well and incubated for 15 to 60 min at 37 °C, 5% CO2). Cells were washed in 100 μl ice-cold PBS and stained for flow cytometry or lysed for western blotting.

### Inhibitors of L-selectin proteolysis

D1(A12) ADAM17 antibody was obtained from Professor Gillian Murphy (Cambridge University) and used at a concentration of 300 nM and human IgG was used as control. The hydroxamate-based ADAM/MMP MP inhibitor Ro 31-9790 was obtained originally from Roche and used at a final concentration of 30 μM. The γ-secretase inhibitors L-685 (5*S*)-(*tert*-Butoxycarbonylamino)-6-phenyl-(4*R*)-hydroxy-(2*R*)-benzylhexanoyl)-L-leucy-L-phenylalaninamide; Sigma-Aldrich) and N-[N-(3,5-difluoro-phenacetyl)-L-alanyl]-S-phenylglycine t-butyl ester, (ALX-270-416) (Enzo, Life Sciences) were both used at 10 μM. Proteasomal inhibitor MG132 (carbobenzoxy-L-leucyl-L-leucyl-L-leucine) was used at 20 μM. MOLT-3 T cells in R1 medium or MEF cells in D1 medium were preincubated for 1 h at 37 °C with inhibitors or equivalent levels of DMSO solvent control at the specified concentrations. L-selectin shedding was stimulated in the presence of the inhibitors or DMSO solvent control as indicated in the figures.

### Lysosomal and autophagy inhibitors

Cells were preincubated for up to 6 h prior to PMA stimulation with the following inhibitors: bafilomycin A1 which prevents autophagosome-lysosome fusion and deacidifies intracellular compartments (100 nM; Sigma-Aldrich), chloroquine which prevents autophagosome-lysosome fusion (20 μM; Sigma-Aldrich), leupeptin and pepstatin lysosomal protease inhibitors (both 20 μM; Sigma-Aldrich), or the autophagy inhibitor MRT68921 (MRT), which inhibits ULK-1 and ULK-2 (1 μM, Sigma-Aldrich) or DMSO vehicle as control prior to addition of 300 nM PMA in the presence of inhibitors for 30 to 45 min.

### Analysis of L-selectin proteolysis by western blotting

L-selectin-V5/His positive 1 × 10^6^ MOLT-3 cells and adherent MEFs or Jurkat T cells expressing endogenous (non-V5–tagged) L-selectin were washed in ice-cold PBS and lysed for 30 min in 35 μl RIPA buffer (25 mM Hepes at pH 7.4, 150 mM sodium chloride, 10 mM magnesium chloride, 1 mM EDTA, 2% glycerol, 1% Triton X-100) supplemented with 4 mM 1,10 phenanthroline, 1 mM sodium orthovanadate, and 1 protease inhibitor tablet per 10 ml of lysis buffer (Roche, Complete protease ULTRA). Cell lysates were centrifuged (5 min, 4 °C, 250*g*), and the supernatants were collected and stored at −20 °C or used immediately. In some experiments 1 × 10^6^ MOLT-3 were lysed in 35 μl Laemmli buffer (660 mM Tris–HCl, pH 6.8, 26% glycerol (v/v), 4% SDS (w/v), 0.01% bromophenol blue (w/v), and 5% β2-mercaptoethanol (v/v). Cell lysates were sonicated on ice for 15 s using a Sonic Dismembrator model-120 (Thermo Fisher Scientific) and centrifuged at 13,000 rpm. Collected supernatants were immediately used for SDS-PAGE and Western blot analysis. Protein concentrations were determined using the Pierce BCA protein assay (Thermo Fisher Scientific).

Lysates were mixed with equal volume of Laemmli sample buffer, heat-denatured and loaded onto 4 to 10% gradient gels (Bio-Rad), and resolved at 100 V. Proteins were transferred to activated polyvinylidene difluoride (PVDF) transfer membrane (methanol, 30 s, RT) with a pore size of 0.2 μm (Thermo Fisher Scientific; 88520) for the capture of small proteins using the X-cell module (60 min, 30 V) according to the manufacturer’s protocol with NuPage transfer buffer (Thermo Fisher Scientific; NP0006).

Membranes were blocked with 5% milk powder in PBS/0.1% Tween 20 (PBS-T) for 1 h at RT prior to incubation with mouse monoclonal anti-V5 (R960-25, Invitrogen; 1 in 1000) or CA21 L-selectin cyto tail antibody (a kind gift from Julius Kahn, Boehringer-Ingelheim; 1/400) (58) overnight at 4 °C with rocking. The following day, the PVDF membrane was washed 5x in PBS-T (5 min, RT, rocking) before addition of the secondary horseradish peroxidase–conjugated antibody (Bio-Rad; 1706516; 1.6/1000 in 5% milk PBS-T; 60 min, RT, rocking). The PVDF membrane was washed 5x in PBS-T (5 min, RT, rocking) before development of the membrane using a chemiluminescent substrate (Thermo Fisher Scientific; 34580) and imaged using a Syngene G:Box or iBright 1500. Following imaging, PVDF membranes were stripped according to manufacturer’s protocol (Thermo Fisher Scientific, 21,059; 15 min, 37 °C) before washing 5x in PBS-T (5 min, RT, rocking), blocking with 5% milk in PBS-T, and reprobing for GAPDH using anti-GAPDH (Abcam, ab9484, 0.32/1000) as primary antibody. For long-term storage, PVDF membranes were sealed in bags with PBS-T.

### Conventional flow cytometry

For surface staining, cells were washed in PBS 2% FCS, labeled with Live/Dead Fixable Aqua (Life Technologies), washed in PBS 2% FCS, and blocked with PBS 2% FCS containing 5% rat serum. Cells were stained with relevant antibodies for 30 min at 4 to 8 °C, washed in PBS 2% FCS, fixed with 4% paraformaldehyde, and resuspended after a wash in 200 μl PBS 2% FCS. Cells were analyzed on a BD FACSCanto II machine using installed DIVA software and data analyzed using FlowJo V10 (https://www.flowjo.com/flowjo10/download). UltraComp eBeads (ThermoFisher) were used following manufacturer’s protocols for compensation during flow cytometry analysis. T cells were stained using anti-CD62L-PE (DREG56) with isotype control P3.6.2.8.1-PE (IgG1κ) (eBioscience). T cell-APC mixtures were costained for CD19 using anti-CD19-APC (H1B18) (BD Biosciences) with isotype control 11711-APC (IgG1κ) (R&D Systems) and CD62L on CD19 negative MOLT-3 T cells analyzed. Cell surface L-selectin expression on MOLT-3 T cells was calculated relative to non-TCR stimulated cells after subtracting MFI or % positive cells for isotype control antibody (Ab)-stained cells from all samples:

% cell surface L-selectin = 100 × (MFI of TCR activated T cells – MFI isotype control)/(MFI unactivated T cells – MFI isotype control)

### Imaging flow cytometry

T cells were surface stained as for conventional flow cytometry and washed once in PBS prior to fixation in 2% fresh formaldehyde (2% v/v in PBS; 60 min, RT; Pierce; 28906). Following this, cells were washed three times in 200 μl True Nuclear permeabilization buffer (BioLegend; 424401) before staining with 50 μl of diluted antibody (again in permeabilization buffer; 30 min, RT). Cells were then washed three times in 200 μl True Nuclear permeabilisation buffer. For nuclear staining, cells were centrifuged (5 min, 4 °C, 1200 rpm) and 15 μl NucBlue (Invitrogen; R37605) was added to each sample (20 min, RT). Otherwise, cells were resuspended in 15 μl fluorescence activated cell sorting buffer. T cells were stained with the following antibodies: CD62L-APC/Fire 750 (DREG56, IgG1κ; BioLegend), CD69-PE (FN50, IgG1κ; Biolegend), V5-AF647 (AB2532221, IgG2a; Invitrogen), and CD107a-FITC (H4A3, IgG1κ Invitrogen). Prior to analysis, cells were transferred to eppendorf tubes, wrapped in foil and stored (4 °C) until analysis. Data acquisition was conducted at a 60x magnification using an ImageStream X Mark II imaging flow cytometer. During acquisition singlets were exported for data analysis. Controls for ImageStream analysis were created through single stains of each antibody and relevant isotypes on live cells and following incubation at 95 °C for 5 min for LIVE/DEAD stain. Analysis was performed using IDEAS software. The imaging flow cytometry software package uses a log-transformed Pearson’s correlation coefficient to evaluate the similarity in the distribution of fluorescence between two probes within user-defined subcellular compartments or “masks.” The software reports this as a “similarity score.” The spot counting feature within IDEAS software was used to distinguish bright regions of a cell from background fluorescence using a user-defined spot-to-cell background in user-defined masks.

### Soluble L-selectin

Soluble L-selectin was quantitated by ELISA (Human L-selectin/CD62L DuoSet ELISA, DY728, R&D systems) according to manufacturer’s instructions and absorbance measured immediately at 450 nM using a CLARIOstar microplate reader. The detection limit was 0.078 ng/ml.

### Statistical analysis

All statistical analyses were conducted using GraphPad Prism 10.0.3 (https://www.graphpad.com/features). Data were tested for normality using the Shapiro–Wilk test. Statistical tests used are presented in each figure legend. *P* values < 0.05 were deemed significant. In each graph or plot, either the individual values or mean are shown, or the mean and SD. Fisher’s least significant difference (LSD) test is abbreviated in figure legends as Fisher’s LSD. Each experiment contains data from three independent experiments (n = 3).

## Data availability

All data are contained within the manuscript.

## Supporting information

This article contains supporting information.

## Conflict of interest

The authors declare that the research was conducted in the absence of any commercial or financial relationships that could be construed as a potential conflict of interest.
