# Supplementary material for: TCR activation stimulates regulated intramembrane proteolysis of L-selectin by presenilin 1 and localized proteasomal degradation of the cytoplasmic tail
Source: J Biol Chem. 2025 Jul 10;301(8):110473. doi: 10.1016/j.jbc.2025.110473 (PMC12355078; doi:10.1016/j.jbc.2025.110473)
Supplement: Supporting information [file mmc1.docx]

**TCR activation stimulates regulated intramembrane proteolysis of L-selectin by presenilin 1 and localized proteasomal degradation of the cytoplasmic tail**

Owen R Moon^1^, Andrew C Newman^1^, Abdullah S Alanazi^1^, Sophie C Wehenkel^1^, Katarzyna Gawel-Bęben^2^. Aleksandar Ivetic^3^, David, A Price^1,4^, Vera Knäuper^2^ and Ann Ager^1,4^

^1^Division of Infection and Immunity, School of Medicine, Cardiff University, Cardiff CF14 4XN, UK

^2^School of Dentistry, Cardiff University, CF14 4XY, UK

^3^Faculty of Life Sciences and Medicine, School of Cardiovascular Medicine and Metabolic Sciences, King’s College London, London SE5 9NU, UK

^4^Systems Immunity Research Institute, Cardiff University, Cardiff CF14 4XN, UK

Owen Moon and Andrew Newman are first authors

Figure S1. Flow cytometry gating strategy and PMA time course and dose response.

Figure S2. ADAM17 dependent membrane retained fragment of endogenous wild type L-selectin is cleaved by γ-secretase dependent.

Figure S3. Definition of membrane and intracellular masks for Imaging Flow Cytometry analysis.

Figure S4. Imagestream Gating strategy to analyse CD62L-V5 tail localisation

Page S-1

**
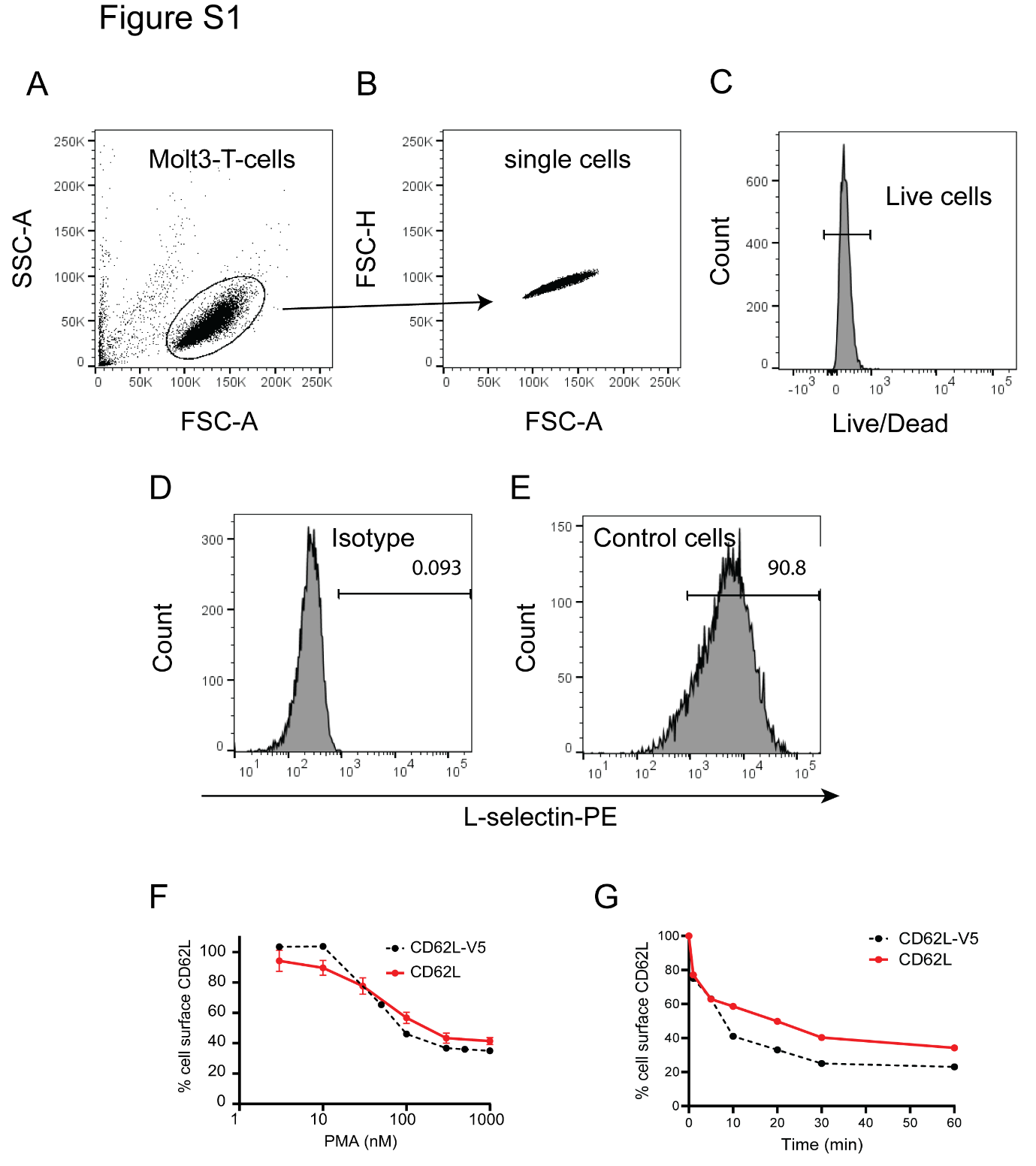
**

**Figure S1. Flow cytometry gating strategy and PMA time course and dose response.** Gates show 868 TCR+ MOLT-3 cells (A) gated for single (B), live cells (C) and stained with isotype control (D) or PE conjugated anti-CD62L (E) antibody. Results are presented either as frequency of L-selectin+ cells (bar) or median fluorescence intensity (top right). 868 TCR+ MOLT-3 cells expressing non-tagged (CD62L) or tagged (CD62L-V5) were incubated with PMA at 0-1000 nM for 30 min PMA (F) or at 300 nM for 0-60 min (G). The frequency of cells expressing cell surface CD62L/L-selectin is shown. Results are mean + SD, n = 3.

Page S-2

**
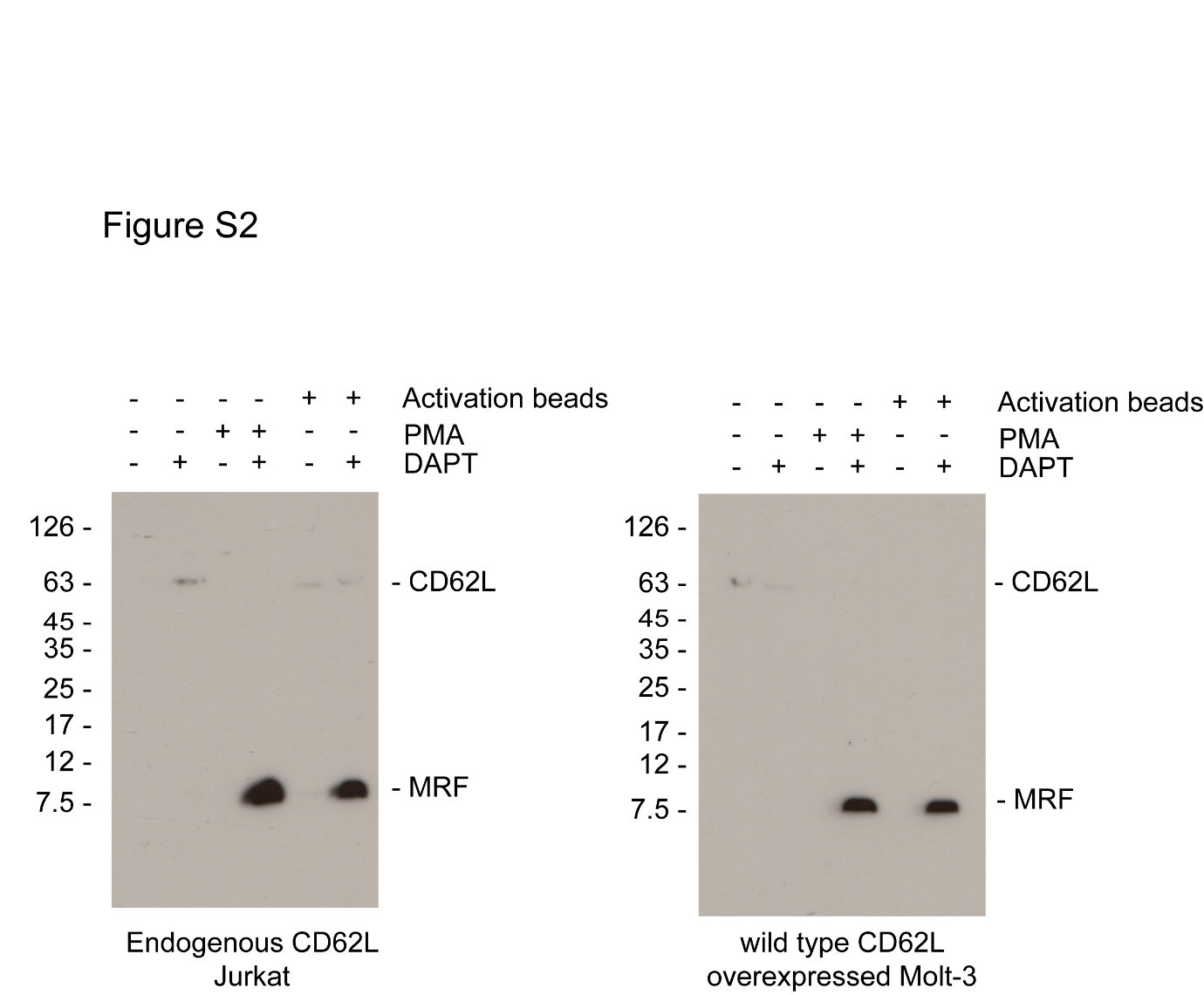
**

**Figure S2. ADAM17 dependent membrane retained fragment of endogenous wild type L-selectin is cleaved by γ-secretase dependent.** Jurkat T cells expressing endogenous L-selectin(A) and 868 TCR+ MOLT-3 cells expressing wild type L-selectin (B) were pretreated with the γ-secretase inhibitor DAPT, stimulated using either PMA or T cell activation beads and cell lysates analysed for full length CD62L and MRF.

Page S-3


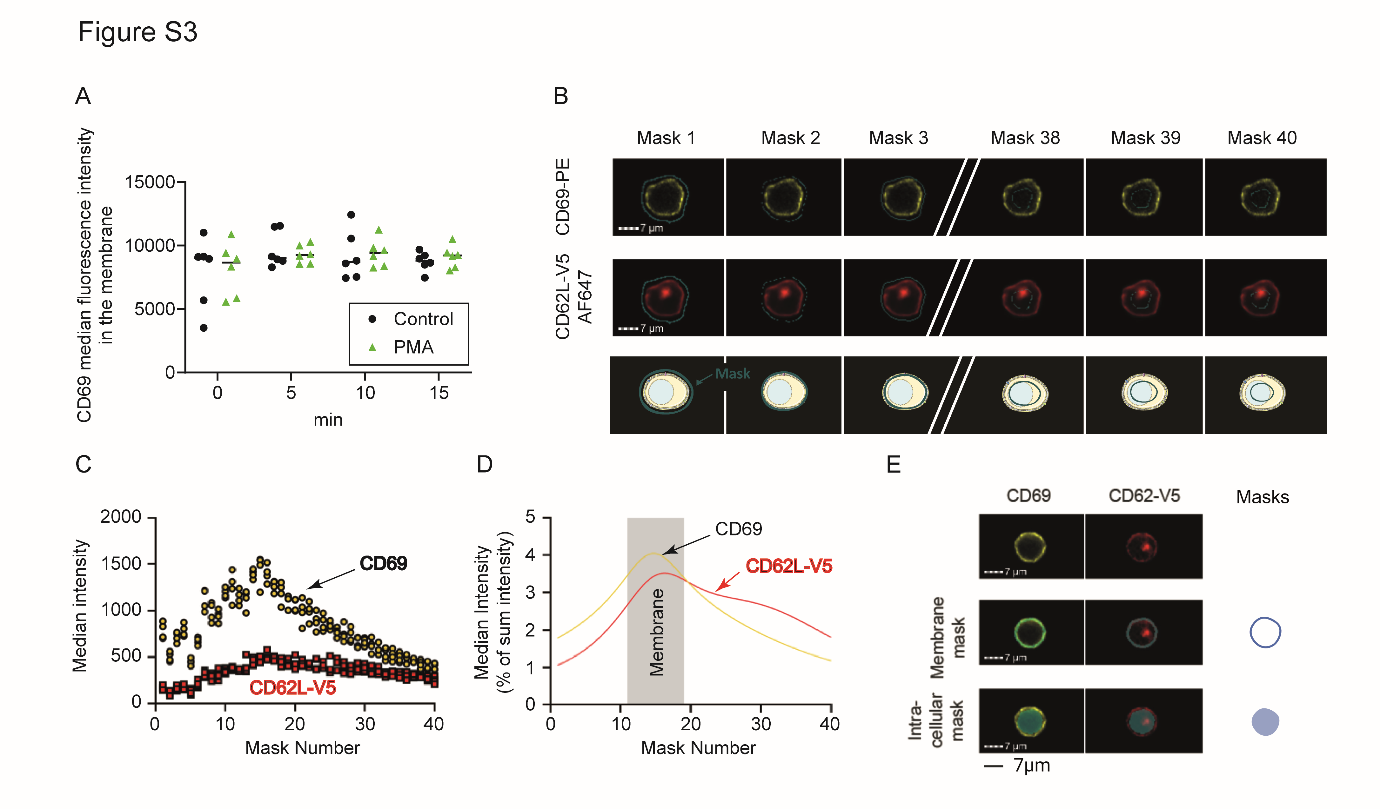


**Figure S3. Definition of membrane and intracellular masks for Imaging Flow Cytometry analysis.** (A) 868 TCR+ CD62L-V5+ MOLT-3 were incubated with PMA or solvent control for up to 15 min and live cells stained and analysed for CD69 expression using Imagestream. Data points show membrane levels of CD69 in the absence and presence of PMA in replicate experiments. Bars represent means + SD, n = 6. Two-way ANOVA with Fisher’s LSD test. (B) Representative images of a single MOLT-3 T cell stained for cell surface CD69 (yellow) and intracellular CD62L-V5 tag (red) and overlaid with a series of concentric masks (blue) generated using the analysis software. Masks one pixel thick are defined and median fluorescence intensity within each ring were extracted. Mask 1 is at the outermost cell periphery and Mask 40 at the cell centre. (C) Median fluorescence intensity within each mask plotted against mask number for CD69 and V5 signals. (D)The normalised values were used to plot the sum of two Lorentzian curves where the width of the first peak approximates to the fluorescence originating from the membrane (grey bar). Mask values within the membrane region (masks 11-19) were pooled to generate a membrane mask which was subtracted from the whole cell mask to give an intracellular mask. (E) Representative image of a membrane positive cell with and without defined membrane and intracellular masks.

Page S-4

**
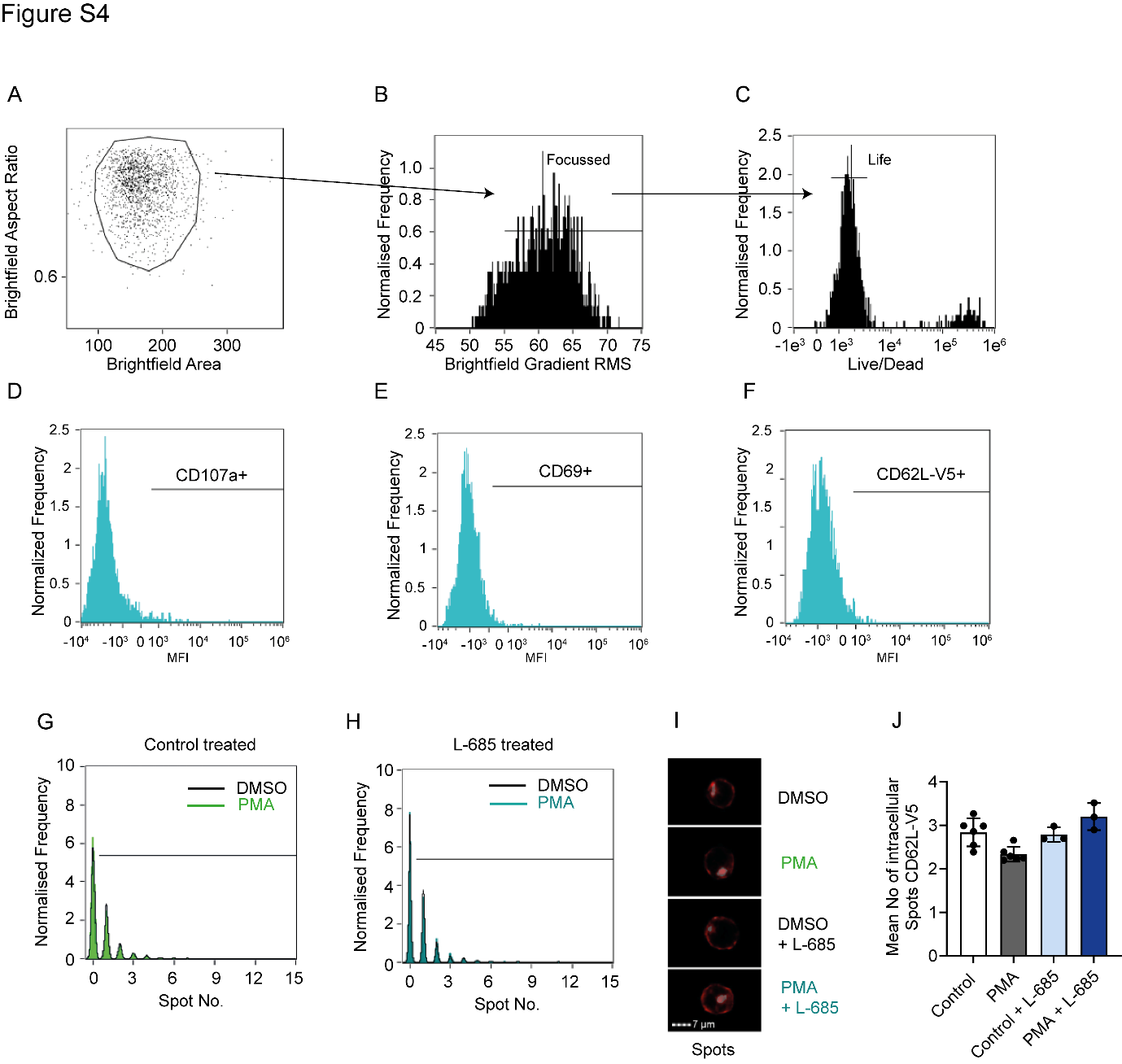
**

**Figure S4. Imagestream Gating strategy to analyse CD62L-V5 tail localisation.** (A-F) Gating strategy used to analyse 868 TCR+ CD62L-V5+ MOLT-3 single (A), focussed (B), live (C) cells by imaging flow cytometry for localisation of the V5 tag in the membrane or intracellular compartments. Isotype controls were used to set gates for lysosome (D) membrane (E) and CD62L-V5 tail (F). (G-J) 868 TCR+ CD62L-V5+ MOLT-3 cells were pre-treated with L-685 or solvent control and incubated with PMA or DMSO solvent control for 15 min and CD62L-V5+ cells were analysed for intracellular spots. The mean number of spots per cell in control (G) and L-685 treated (H) cells, representative images of spot+ cells (I) and the mean number of CD62L-V5 + spots (J) in the different treatment groups are shown. Data are means + SD, n=6. One way ANOVA.

Page S-5
